# Decoding individual natural scene representations during perception and imagery

**DOI:** 10.3389/fnhum.2014.00059

**Published:** 2014-02-12

**Authors:** Matthew R. Johnson, Marcia K. Johnson

**Affiliations:** ^1^Department of Psychology, Yale UniversityNew Haven, CT, USA; ^2^Interdepartmental Neuroscience Program, Yale UniversityNew Haven, CT, USA

**Keywords:** visual imagery, visual perception, MVPA, fMRI, classification, decoding, scene, PPA

## Abstract

We used a multi-voxel classification analysis of functional magnetic resonance imaging (fMRI) data to determine to what extent item-specific information about complex natural scenes is represented in several category-selective areas of human extrastriate visual cortex during visual perception and visual mental imagery. Participants in the scanner either viewed or were instructed to visualize previously memorized natural scene exemplars, and the neuroimaging data were subsequently subjected to a multi-voxel pattern analysis (MVPA) using a support vector machine (SVM) classifier. We found that item-specific information was represented in multiple scene-selective areas: the occipital place area (OPA), parahippocampal place area (PPA), retrosplenial cortex (RSC), and a scene-selective portion of the precuneus/intraparietal sulcus region (PCu/IPS). Furthermore, item-specific information from perceived scenes was re-instantiated during mental imagery of the same scenes. These results support findings from previous decoding analyses for other types of visual information and/or brain areas during imagery or working memory, and extend them to the case of visual scenes (and scene-selective cortex). Taken together, such findings support models suggesting that reflective mental processes are subserved by the re-instantiation of perceptual information in high-level visual cortex. We also examined activity in the fusiform face area (FFA) and found that it, too, contained significant item-specific scene information during perception, but not during mental imagery. This suggests that although decodable scene-relevant activity occurs in FFA during perception, FFA activity may not be a necessary (or even relevant) component of one's mental representation of visual scenes.

## Introduction

Current models of working memory and related reflective activities (e.g., mental imagery) suggest that active representations are maintained via control signals originating in heteromodal association areas (e.g., prefrontal cortex) that re-instantiate neural activity in sensory cortex that was first engaged when an item was initially perceived (Petrides, 1994; Kosslyn et al., 2001; Curtis and D'Esposito, 2003; Ruchkin et al., 2003; Pasternak and Greenlee, 2005; Ranganath and D'Esposito, 2005). Consistent with these models, earlier neuroimaging studies observed category-related activity in category-selective extrastriate (CSE) visual areas such as fusiform face area (FFA; Kanwisher et al., 1997; McCarthy et al., 1997) and parahippocampal place area (PPA; Epstein and Kanwisher, 1998) when individuals maintained representations of items from the relevant category during visual working memory (Druzgal and D'Esposito, 2003; Postle et al., 2003; Ranganath et al., 2004). Similar category-specific activity is also seen during visual mental imagery (O'Craven and Kanwisher, 2000) and in response to shifts of reflective attention toward a particular active representation (e.g., *refreshing*; Johnson et al., 2007; Lepsien and Nobre, 2007; Johnson and Johnson, 2009).

Such studies, however, provide only circumstantial evidence supporting the idea that category-specific activity in CSE cortex reflects information about the identity of individual item representations. An alternative explanation is that thinking of items from a particular category causes a general increase in baseline activity in relevant CSE areas, without that activity containing any information about the specific item from that category being held in mind. For example, one study (Puri et al., 2009) found that preparation to view faces or houses induced greater activity in FFA and PPA, respectively, even though participants only knew which category to expect rather than any particular exemplar from the category. In order to determine that item-specific information is also present in reflection-induced activity, a method is needed that is capable of assessing cortical activation patterns related to individual items within a category, when those items' representations are presumed to involve similar overall category-specific activity increases in CSE cortex. Multi-voxel pattern analysis (MVPA) is one method that can assess such patterns.

A number of studies in recent years have used MVPA to directly probe how information is represented in visually responsive brain areas. Several initial studies focused on classifying general categories of items during visual perception, finding that information about the category being viewed could be reliably decoded in many visually responsive cortical regions (Haxby et al., 2001; Cox and Savoy, 2003; Norman et al., 2006). Pattern analyses have also been used to decode category information during working memory maintenance (Han et al., 2013) or visual imagery (Cichy et al., 2012), and pattern analysis may afford better detection of category-related brain activity due to reflective processing than more traditional univariate functional magnetic resonance imaging (fMRI) analyses (Han et al., 2013).

Following reports of successful category classification, there has been increasing interest in using MVPA to decode more fine-grained information in visually responsive brain regions, at the sub-category or exemplar levels. [The terminology varies in published papers, but here we use the term “category” to refer to stimulus classes such as faces, scenes, objects, and body parts that are associated with known CSE regions such as FFA, PPA, lateral occipital complex (LOC), and extrastriate body area (EBA), respectively; “sub-category” to refer to smaller groupings such as “forests” vs. “mountains” within the category “scenes” or “tools” vs. “fruits” within the category “objects”; and “exemplar” to refer to individual items within a category or sub-category.] Multi-voxel classification analyses have revealed exemplar-specific activity during visual perception in LOC for objects (Eger et al., 2008) and anterior inferior temporal cortex for faces (Kriegeskorte et al., 2007). Other studies have been able to construct reliable predictions of the visual stimulus being projected onto the retina based on activity in early visual cortex (Kay et al., 2008; Miyawaki et al., 2008).

Several studies also successfully used classification techniques to decode information at the sub-category or exemplar level during working memory maintenance or visual imagery. Activity in early visual cortex, LOC, and other areas has been used to predict the identity or characteristics of simple stimuli, such as the orientation or contrast of gratings, or X's vs. O's (Thirion et al., 2006; Harrison and Tong, 2009; Serences et al., 2009; Stokes et al., 2009; Xing et al., 2013). For more complex stimuli, Reddy et al. (2010) were able to decode the object sub-categories of tools and food (as well as buildings and faces) during both perception and mental imagery, based on activity in a large set of face-, scene-, and object-responsive voxels. More recently, Lee et al. (2012) were able to decode the identities of individual object exemplars (e.g., a bag, a car, a chair) without regard to possible sub-category groupings during perception and imagery, based on activity in LOC as well as retinotopic visual areas.

The studies cited above provide broad support for the general notion that multiple visually responsive brain areas represent information about not only the overall category, but also the sub-category, characteristics, or identity of specific items maintained in working memory/visual mental imagery during reflective processing. However, there remain many open questions regarding what type of information is represented in which brain areas for a given item or category, and whether the nature or quality of that information differs between perceptual processing and reflective (working memory/mental imagery) processing. The research landscape regarding the brain's representation of natural visual scenes is particularly complex, given the wide variety of possible visual scenes, the many ways in which they can be characterized or sub-categorized, and the large number of scene-responsive brain regions.

For the visual perception of natural scenes, Walther et al. (2009) found that PPA and retrosplenial cortex (RSC) did encode information distinguishing different sub-categories of scenes in a block design during perception, and Kriegeskorte et al. (2007) also found that PPA distinguished between two house pictures used in that study. Park et al. (2011) found via MVPA that PPA, RSC, and other areas distinguished between scenes with urban vs. natural content, and between scenes with closed vs. open spatial boundaries; and Epstein and Morgan (2012) found that several scene-responsive regions contained information distinguishing not only scene sub-categories, but the identities of different specific visual landmarks. Bonnici et al. (2012) also found that activity patterns in the medial temporal lobe could be used to distinguish between highly visually similar scenes.

However, to our knowledge, no study to date has used pattern analysis to examine item-specific information in any visual area during working memory or mental imagery for natural scenes. Thus, the primary aim of the present study was to determine if activity in scene-selective areas of cortex represents item-specific information during mental imagery, and to what extent that information constitutes a re-instantiation of item-specific activity patterns observed during visual perception.

In this study, we presented participants with either pictures of previously memorized scenes to view, or with verbal labels of those pictures, in which case participants were instructed to remember and form the most vivid and accurate mental image possible of the indicated picture. A face-scene localizer task allowed us to locate several scene-selective regions of interest (ROIs), and then we used MVPA to assess whether those areas reliably encoded information about the identity of specific scene items during perception and/or imagery. We also examined whether item-specific activity patterns from perception were re-instantiated during mental imagery.

Based on previous reports that different scene-selective areas may participate to different degrees in top-down vs. bottom-up representations of visual scenes (e.g., Johnson et al., 2007), we also used MVPA to test whether all scene-selective areas reliably distinguished between the overall processes of visual perception and mental imagery, and to what extent the ability to differentiate between perception and imagery differed by region.

Finally, this experimental design also allowed us to localize the FFA and address a secondary question, namely whether scene identity information is limited to CSE areas that are maximally selective for scenes, or whether a CSE area such as the FFA could also contain identity information about a category other than the one for which the area is maximally selective.

## Materials and methods

### Participants

Sixteen healthy young adults participated in Experiment 1 [7 females, mean age = 23.1 ± 2.7 (*SD*)]. For Experiment 2, 12 participants (some, but not all, of whom were the same individuals as in the first study) were scanned [7 females, mean age = 23.3 ± 3.0 (*SD*)]. All participants gave written informed consent and were compensated for their time in a protocol approved by the Yale University Human Investigation Committee.

### Task—Experiment 1

**Figure 1:**
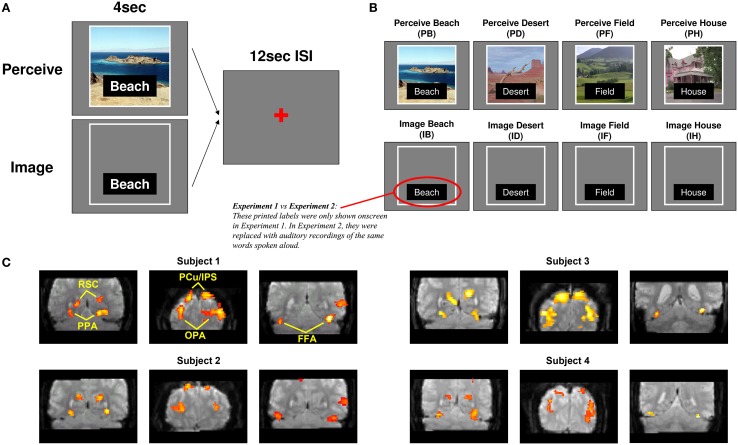
**Task design. (A)** On *Perceive* trials, participants were shown a picture of a scene along with its label for 4 s. On *Image* trials, participants saw only an empty frame with a label instructing which of the four scenes to imagine. The example displays shown here correspond to Experiment 1; in Experiment 2, the displays were the same except that the printed labels were removed entirely and replaced with auditorily presented recordings of the same words spoken aloud. **(B)** The two processes (*Perception, Imagery*) × the 4 stimuli (*Beach, Desert, Field, House*) formed a total of 8 conditions of the task. **(C)** Sample ROI locations for four representative subjects, two from Experiment 1 and two from Experiment 2. Clusters are overlaid on raw functional images from that participant's data.

The version of the main Perception-Imagery (P-I) task used in Experiment 1 is shown in Figure 1. Before fMRI scanning, participants repeatedly viewed four scene pictures (for all participants, a *beach*, a *desert*, a *field*, and a *house*) and were instructed to memorize the details of the pictures as well as they could for later mental imagery. For the P-I task (Figure 1A), on each trial, participants were either shown one of the pictures along with its name (*Perception*) or simply the name of one picture (*Beach, Desert, Field*, or *House*), in which case they were instructed to form the most vivid and accurate mental image possible of that picture as long as the label was onscreen (*Imagery*). Thus the 2 processes (*Perception, Imagery*) × the 4 stimuli (*Beach, Desert, Field, House*) formed a total of 8 conditions [*Perceive Beach* (PB), *Image Beach* (IB), *Perceive Desert* (PD), and so on] of the task (Figure 1B). These four scene pictures were intentionally selected from different sub-categories of visual scenes with relatively large differences in color, spatial composition, etc., to minimize featural confusion between images. Thus successful classification between items in this study would likely reflect information differences at some combination of the sub-category and exemplar (within sub-category) levels, somewhat limiting the granularity of information representation that could be deduced but also maximizing chances of successful classification, while using a design that could easily be extended in future studies to examine more fine-grained differences among scene exemplars (see Discussion). In this paper, we will refer to the different scenes used simply as “items” and information revealed in classification as “item-specific,” acknowledging that such information likely comprises a fusion of sub-category-specific and exemplar-specific information.

Pictures or labels were onscreen for 4 s each with an inter-trial interval of 12 s. The pictures occupied approximately 20 degrees of visual angle. Conditions were presented in a pseudo-random order optimized to produce maximal orthogonality between conditions during subsequent fMRI analyses. To counterbalance trial orders across participants, every participant encountered the runs of the task in a different order, and for every second participant perception and imagery trials were switched. Participants practiced the task both before scanning and during the anatomical scans that occurred immediately prior to functional scanning, in order to ensure that their memories of the stimuli were fresh and to increase the likelihood that any repetition attenuation effects from repeatedly viewing the same stimuli would have reached asymptote by the time functional scans began.

### Task—Experiment 2

Although scene-selective areas such as PPA are not typically sensitive to non-scene stimuli (e.g., letter strings), it is theoretically possible that the minor visual differences between words used to cue the item to imagine (e.g., “Desert,” “Field”; see Figure 1) could result in successful classification between items on mental imagery trials, rather than the mental images themselves. To confirm that this was not the case, we conducted a replication (Experiment 2) in which 12 participants performed the same P-I task as in Experiment 1, except that the visual labels of the pictures were removed from both Perception and Imagery trials and replaced by auditory labels [recordings of a male voice speaking the same words as the visual labels (*Beach, Desert, Field, House*)]. Auditory labels were presented via headphones at the beginning of each (Perception or Imagery) trial. All other aspects of the study were identical between Experiments 1 and 2.

### fMRI data acquisition

Scanning was performed on a Siemens 3T Trio system with a standard 8-channel head coil. Functional scans consisted of a moderately high-resolution (2 × 2 × 2.5 mm) echoplanar imaging sequence (parameters: *TE* = 24 ms, flip angle = 60°, FoV = 256 mm, FoV phase = 75%, interleaved acquisition, 26 slices, *TR* = 2000 ms). Participants performed 6 functional runs of the P-I task. Each run lasted 8 min 50 s (265 volumes) and contained 32 trials (4 per condition), for a total of 24 trials per condition per participant. The first 6 volumes (12 s) of each run were discarded to allow time for the fMRI signal to reach steady state. As these scan parameters did not allow for whole-brain coverage, slices were manually prescribed at an oblique angle based on visual inspection of the participant's head shape after initial anatomical scans were acquired. Slices were tilted at the angle deemed most likely to provide coverage of the four major scene-selective ROIs noted below (based on the average locations of these ROIs from previous group analyses of localizer tasks).

### Statistics and data analysis

Initial processing of fMRI data was performed using SPM5 (Wellcome Department of Imaging Neuroscience, University College London, UK). Data were motion-corrected, and all of a participant's functional runs were coregistered to a mean image of that participant's first run after motion correction. Prior to classification, an initial general linear model (GLM) was estimated for each participant's data from the P-I task as a means of essentially collapsing fMRI signal from the multiple functional volumes acquired in each trial into a single volume. In this GLM analysis, each individual trial of the task (defined as an event with 4 s duration) was convolved with a canonical hemodynamic response function, producing a separate regressor in the model for each trial. Estimating this GLM [using an autoregressive AR(1) model to remove serial correlations during estimation] produced a volume of beta values for each trial of the P-I task, representing overall activation in each voxel of the brain for that trial. Each beta image was transformed into Z-scores to control for any differences in overall brain activation between trials. Values from these Z-transformed beta images were used as the basis for classification analyses (see below). Classification analyses on the main P-I task were all performed on unsmoothed data.

For each subject, scene-selective ROIs were selected using a face-scene localizer task similar to that used in previous studies (Wojciulik et al., 1998; Yi and Chun, 2005; Johnson et al., 2007). Each participant performed 2 runs of this task; each run contained 4 blocks (16 s long) of faces and 4 blocks of scenes. Each block contained 20 stimuli (shown for 500 ms with a 300 ms inter-stimulus interval) presented centrally; blocks were separated by 16 s blocks of rest. Participants were instructed to watch the streams of pictures closely and press a button every time they saw the same picture twice in a row (1-back task). Each localizer run lasted 4 min 24 s (132 volumes) and used the same scan parameters and slice positioning as the main P-I task. Data were motion-corrected in the same manner as the P-I task and were also coregistered to the first run of the P-I task, so that functional data from both tasks were in the same anatomical space. Face and scene blocks were modeled as 16 s events and convolved with the canonical HRF to form regressors for another GLM analysis, and scene-selective ROIs were obtained by assessing the *Scene > Face* contrast from this analysis. [It is worth noting that the “scene-selective” ROIs we discuss here are not necessarily areas that activate *exclusively* for scenes; they are simply scene-selective insofar as they activate preferentially for scenes compared to at least one other category of complex, naturalistic visual stimuli (faces).] However, in contrast to the main P-I task, the same GLM was estimated for both the unsmoothed localizer data and for a second copy of the data that had been smoothed with a Gaussian kernel [5 mm full width at half maximum (FWHM)], for purposes of locating ROIs.

Specifically, scene-selective ROIs were obtained by initially running the above GLM on the *smoothed* functional data from the localizer task and examining the *Scene > Face* contrast (generally at a *p* threshold of 0.001, uncorrected, and a cluster threshold of 10 voxels, although thresholds were relaxed as necessary to locate certain ROIs for a few participants). We located four bilateral ROIs for each participant that had reliably appeared in group analyses of face-scene localizer data in previous studies (Johnson et al., 2007; Johnson and Johnson, 2009): PPA (Epstein and Kanwisher, 1998); RSC (O'Craven and Kanwisher, 2000); an occipital scene area which has been variously referred to as the transverse occipital sulcus (TOS; Grill-Spector, 2003; MacEvoy and Epstein, 2007), middle occipital gyrus (MOG; Johnson et al., 2007; Johnson and Johnson, 2009), or occipital place area (OPA; Dilks et al., 2013; the nomenclature we use here), and an area located near the precuneus/intraparietal sulcus (PCu/IPS; Johnson et al., 2007; Johnson and Johnson, 2009).

**Figure 2:**
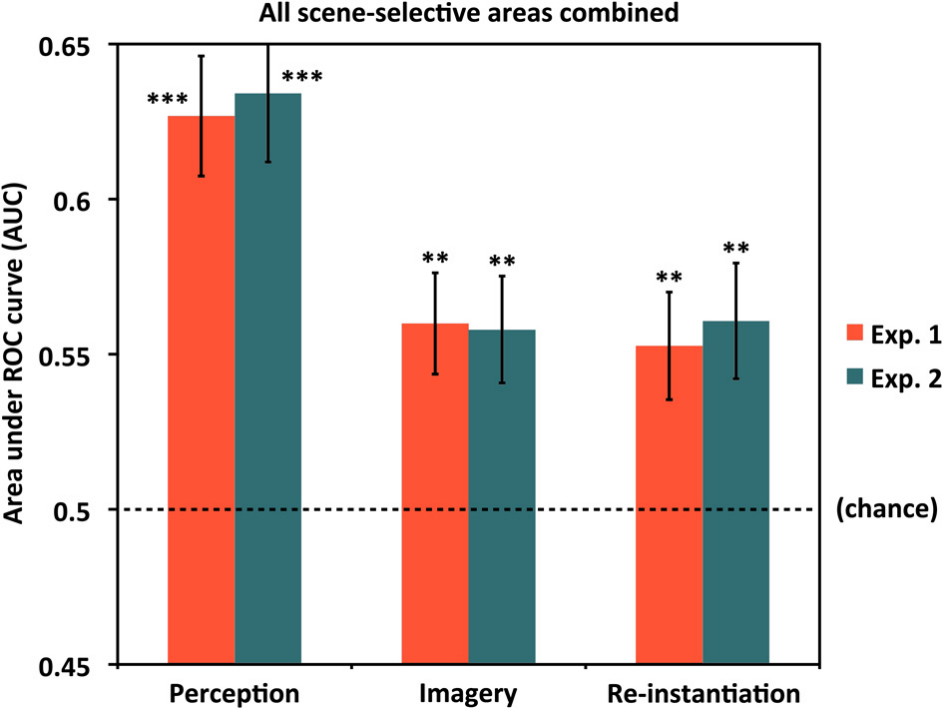
**Classification across all scene areas**. Classification accuracy for Experiments 1 and 2 using voxels from all scene-selective ROIs. Analyses used 640 voxels per participant (4 scene-selective regions × 2 hemispheres × 80 voxels per region). Results are shown for classifying between individual scene items during perception (left bars), classifying between scenes during mental imagery (middle bars), and re-instantiation of perceptual information during mental imagery (right bars). All were significantly above chance (AUC = 0.5) for both experiments. ^**^p < 0.01, ^***^p < 0.001. Error bars represent standard error of the mean (s.e.m.). See text and Table 1 for full statistics.

For each participant, we selected the peak voxel from each cluster corresponding to the approximate anatomical location of these ROIs in prior group analyses, and focused on a 10 mm-radius sphere around that peak voxel for each ROI (examples of all ROIs for four representative participants are shown in Figure 1C). Within each spherical ROI, we then selected only the 80 most scene-selective voxels (approximately 20% of the 410 voxels found in each 10 mm-radius sphere) for classifier analyses, in order to eliminate noise input from voxels that might contain white matter, empty space, or gray matter that was not strongly activated by scene stimuli (for one participant at one ROI, only 65 in-brain voxels were found within 10 mm of the peak voxel of that ROI, so only those 65 voxels were used). This 80-voxel figure was initially chosen as an informed estimate of the number of “good” gray matter voxels that could be expected to be contained in each 10 mm-radius, 410-voxel sphere. Subsequent analyses (conducted after the main analyses discussed below, using the *a priori* number of 80 voxels, were completed) compared the results from using 10, 20, 40, 80, 160, or 320 voxels per spherical ROI, and found that classification performance did effectively plateau at around 80 voxels for most ROIs (see Supplementary Figure 1), and in some cases decreased for 160 or 320 voxels relative to 80 voxels. Scene selectivity was assessed by using the *t*-statistic for the *Scene > Face* contrast of the GLM analysis of the *unsmoothed* localizer data. For the classification analyses of individual category-selective ROIs, all of which were found bilaterally for all participants, the 80 voxels from each hemisphere were combined for classification, so a total of 160 voxels were used for each area. For the classification analyses across all scene areas shown in Figure 2 (see Results), voxels from both hemispheres and all four ROIs were fed into the classifier. Thus, the classification across all scene areas shown in Figure 2 used (80 voxels) × (4 ROIs) × (2 hemispheres) = 640 voxels as input.

After voxel selection, Z-transformed beta values from each voxel for each trial were extracted from the GLM analysis of the unsmoothed P-I task data and fed into a support vector machine (SVM) classifier, using custom Matlab code centered around the built-in SVM implementation within Matlab.

### Analyses of item-level information

For analyses of item-level information during perception or imagery, voxels were separated by run and we used a k-fold cross-validation approach, taking data from 5 runs of the P-I task as training data and the remaining run as test data, and then rotating which run was used as test data through all 6 runs of the task (due to time constraints, one participant only had 5 runs of the task; analyses were adjusted accordingly). For each participant, classification results reported in the text and figures were obtained by first training a separate classifier for each pair of conditions (e.g., PB vs. PD, ID vs. IF, and so on), and then applying each classifier to all trials of the test data set (regardless of whether the condition of that trial was one of the ones used to initially train the classifier). Thus, for each pairwise classifier, each trial received a score (either positive or negative, in arbitrary units) indicating the classifier's relative confidence that the trial belonged to one or the other of the conditions used to train it. Then, for each condition, the scores for all trials were collapsed across relevant classifiers (e.g., for condition PB in classifying individual scene items during perception, the scores for the PB vs. PD, PB vs. PF, and PB vs. PH classifiers would be averaged), ultimately yielding a confidence score for each trial and each condition that the trial in question belonged to that condition, relative to all other conditions. These scores were then used to calculate receiver operating characteristic (ROC) curves and the area under the ROC curve (AUC) for each condition and each participant. Finally, AUCs were averaged across condition for each participant to yield a single AUC value for each participant in each analysis (perception, imagery), indicating the algorithm's accuracy at distinguishing among the initially specified conditions for that participant. These AUC values (ranging from 0 to 1, with chance = 0.5) were then subjected to traditional group statistics (e.g., *t*-tests against chance).

### Re-instantiation analyses

To test for evidence of re-instantiation (i.e., similar item-specific neural activity during perception and imagery), we trained a separate group of classifiers similar to the above. However, instead of using k-fold cross validation, these classifiers simply used each possible pair of *Perceive* conditions for all 6 runs as training data (e.g., PB vs. PD, PF vs. PH) and the corresponding pair of *Image* conditions for all 6 runs as test data (e.g., IB vs. ID, IF vs. IH, respectively) to determine whether the same criteria used to classify two items during perception could also classify the same two items during imagery. Relevant classifier scores were collapsed, AUCs were calculated, and statistical tests were conducted as above.

(We also performed a version of this analysis training on *Image* trials and testing on *Perceive* trials, but as the results were virtually indistinguishable from those of training on *Perceive* trials and testing on *Image* trials, only the latter are reported here.)

### Perception vs. imagery analyses

To test for overall classification of perception vs. imagery in each scene-selective ROI, a k-fold cross validation approach was again used as in the analyses of item-level information during perception or imagery. However, classification was much simpler, as each trial was simply coded as either a *Perception* or an *Imagery* trial, and thus only a single (*Perception* vs. *Imagery*) SVM classifier needed to be trained for each fold of the cross-validation. AUCs were calculated and statistical tests conducted as in all other analyses.

### Item-specific information in FFA

For the analyses examining whether face-selective cortex also contained information about the identities of specific scenes, procedures were identical to those outlined above for the scene-selective ROIs, except for the following: The *Face > Scene* contrast was evaluated in the face-scene localizer analysis, we chose clusters located near the known anatomical locations of left and right FFA, and we selected the most face-selective (rather than the most scene-selective) voxels within a 10 mm radius of those clusters' peak voxels.

## Results

Participants performed a task (Figure 1) in which they either perceived or were instructed to form a mental image of one of four previously memorized scene stimuli (a beach, a desert, a field, and a house), yielding a total of eight conditions: *Perceive Beach* (PB), *Image Beach* (IB), *Perceive Desert* (PD), and so on. We examined activity in four scene-selective *a priori* ROIs (OPA, PPA, RSC, and PCu/IPS, as noted in the Materials and Methods section; see Figure 1C), as well as FFA, and used an SVM classification algorithm to determine whether each ROI contained information that allowed the classifier to distinguish between each pair of conditions.

### Classification across all scene areas

Before examining classification performance in individual ROIs, we first examined whether the entire set of scene-selective voxels contained information about individual scene items during perception and/or mental imagery (Figure 2; see Table 1 for *t*-statistics, *p*-values, and effect sizes). We found highly reliable classification between individual scene items during perception (AUCs: Experiment 1 = 0.627, Experiment 2 = 0.634), indicating that scene-selective cortex as a whole did contain item-specific information. Classification between individual scene items during imagery was also above chance (AUCs: Experiment 1 = 0.560, Experiment 2 = 0.558), indicating that scene-selective cortex contains item-specific information during imagery as well. Furthermore, classifiers testing for re-instantiation (i.e., similar item-specific neural activity during perception and imagery, as evidenced by successful classification when using the *Perceive* conditions as training data and *Image* conditions as test data) also performed above chance for scene-selective cortex as a whole (AUCs: Experiment 1 = 0.553, Experiment 2 = 0.561). This confirmed our hypotheses that scene-selective cortex contains information distinguishing individual scene items during both perception and imagery, and that item-specific activity from perception is re-instantiated during mental imagery.

**Table 1:** **Statistical summary of critical results**.

	**Experiment 1**				**Experiment 2**				**Replication**	
**ROI**	**AUC**	***d***	***t***	***p***	**AUC**	***d***	***t***	***p***	***X*^2^**	***p***
**(A) CLASSIFICATION OF ITEM-SPECIFIC SCENE INFORMATION DURING PERCEPTION**										
OPA	0.579	1.06	4.24	0.00071	0.610	1.83	6.33	5.6 × 10^−5^	34.1	7.1 × 10^−7^
PPA	0.598	1.40	5.61	4.9 × 10^−5^	0.583	1.04	3.61	0.0041	30.8	3.3 × 10^−6^
RSC	0.525	0.490	1.96	0.069	0.526	0.587	2.03	0.067	10.8	0.029
PCu/IPS	0.564	1.14	4.56	0.00038	0.548	0.633	2.19	0.051	21.7	0.00023
Combined	0.627	1.64	6.56	9.1 × 10^−6^	0.634	1.75	6.05	8.3 × 10^−5^	42.0	1.7 × 10^−8^
FFA	0.574	1.94	7.75	1.3 × 10^−6^	0.565	0.841	2.91	0.014	35.7	3.4 × 10^−7^
**(B) CLASSIFICATION OF ITEM-SPECIFIC SCENE INFORMATION DURING IMAGERY**										
OPA	0.536	0.566	2.23	0.042	0.554	0.927	3.21	0.0083	15.9	0.0031
PPA	0.529	0.448	1.79	0.094	0.503	0.057	0.20	0.85	5.1	0.28
RSC	0.537	0.806	3.22	0.0057	0.531	0.712	2.47	0.031	17.3	0.0017
PCu/IPS	0.533	0.620	2.48	0.025	0.545	0.618	2.14	0.055	13.1	0.011
Combined	0.560	0.917	3.67	0.0023	0.558	0.970	3.36	0.0064	22.3	0.00018
FFA	0.521	0.386	1.55	0.14	0.503	0.069	0.24	0.82	4.3	0.37
**(C) RE-INSTANTIATION OF ITEM-SPECIFIC INFORMATION FROM PERCEPTION TO IMAGERY**										
OPA	0.517	0.327	1.31	0.21	0.515	0.208	0.72	0.49	4.6	0.34
PPA	0.544	0.680	2.72	0.016	0.536	0.787	2.73	0.020	16.1	0.0028
RSC	0.521	0.411	1.64	0.12	0.524	0.670	2.32	0.040	10.6	0.031
PCu/IPS	0.527	0.670	2.68	0.017	0.525	0.499	1.73	0.11	12.5	0.014
Combined	0.553	0.760	3.04	0.0083	0.561	0.939	3.25	0.0077	19.3	0.00068
FFA	0.523	0.400	1.60	0.13	0.505	0.093	0.32	0.75	4.6	0.33

*All statistics represent two-tailed t-tests against a chance AUC value of 0.5. Replication X^2^ and p-values were obtained by Fisher's method of combining p-values across replications (Fisher, 1925). Experiment 1: all degrees of freedom (df) = 15. Experiment 2: all df = 11. AUC, area under ROC curve; d, Cohen's d*.

### Classifying individual scene representations during perception by ROI

**Figure 3:**
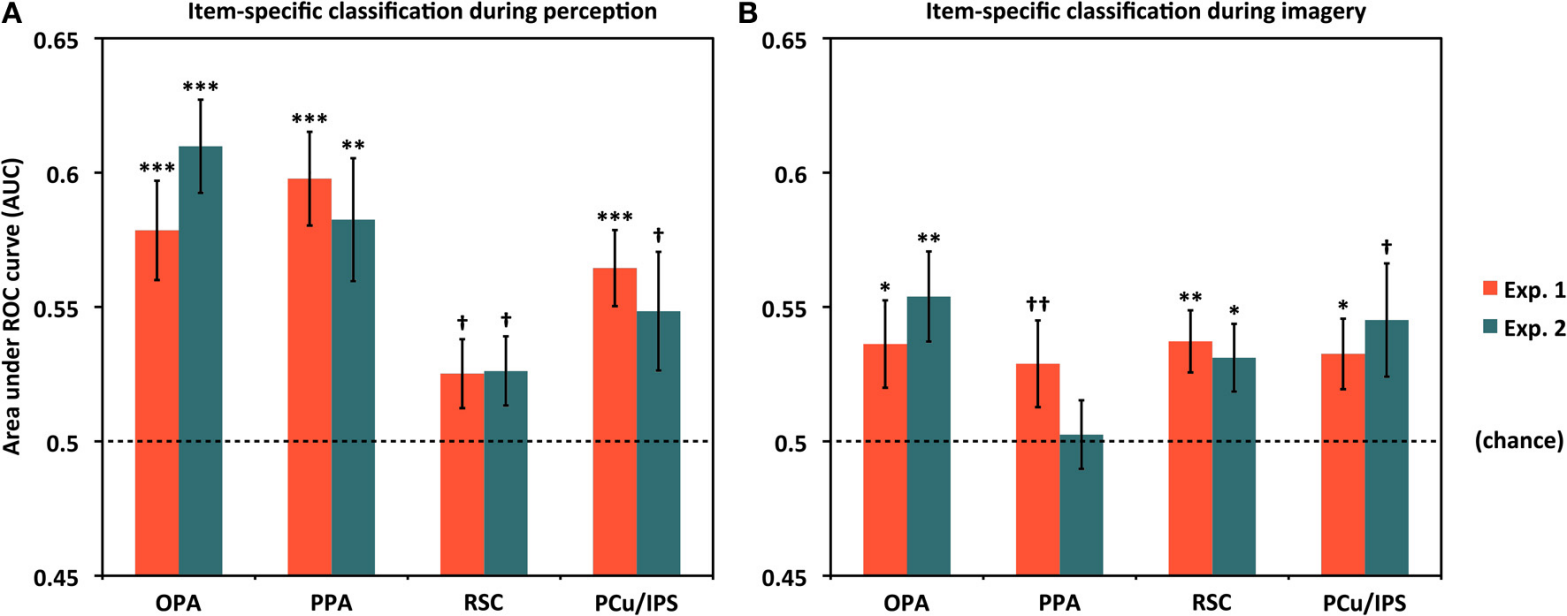
**Classifying individual scenes during perception and imagery by ROI. (A)** Classification accuracy for distinguishing between different scene items during perception for Experiments 1 and 2. In all cases, classification was above chance (AUC = 0.5) either significantly or at a trend level. **(B)** Classification accuracy for distinguishing between different scene items during mental imagery for Experiments 1 and 2. In all cases but PPA in Experiment 2, accuracies were significantly or near-significantly above chance. Analyses used 80 voxels per hemisphere per region, for a total of 160 voxels per region. ^*^p < 0.05, ^**^p < 0.01, ^***^p < 0.001, ^†^*p* < 0.07, ^††^*p* < 0.10. Error bars represent s.e.m. See text and Table 1 for full statistics.

Having shown that item-specific information is present in scene-selective cortex broadly construed, we then performed follow-up tests examining whether above-chance classification could be observed in individual ROIs. Results for item-specific classification in each ROI are shown in Figure 3A and Table 1A. As fewer voxels were being fed into the classifier, performance in individual ROIs might be expected to be lower and more variable than for all scene-selective areas combined. Nevertheless, for perception, we found above-chance classification significantly or at a trend level in all four ROIs in Experiment 1 [AUCs: OPA = 0.579, PPA = 0.598, RSC = 0.525 (*p* = 0.069), PCu/IPS = 0.564] and Experiment 2 [AUCs: OPA = 0.610, PPA = 0.583, RSC = 0.526 (*p* = 0.067), PCu/IPS = 0.548 (*p* = 0.051)]. These findings suggest that all of the scene-selective extrastriate areas we examined contained information distinguishing between individual natural scenes during perception.

### Classifying individual scene representations during imagery by ROI

We next tested whether above-chance scene classification could also be observed in individual scene-selective ROIs during mental imagery (Figure 3B and Table 1B). Classification performance during imagery was generally lower than for perception, as expected, but still above chance significantly or at a trend level in all of our ROIs in Experiment 1 [AUCs: OPA = 0.536, PPA = 0.529 (*p* = 0.094), RSC = 0.537, PCu/IPS = 0.533] and in three out of four ROIs in Experiment 2 [AUCs: OPA = 0.554, PPA = 0.503 (n.s.), RSC = 0.531; PCu/IPS = 0.545 (*p* = 0.055)]. This suggests that the scene-selective areas in OPA, RSC, and PCu/IPS all contained information distinguishing between individual natural scenes during reflective acts such as mental imagery as well as during perception. In PPA, classification was only marginally above chance in Experiment 1 and did not differ significantly from chance in Experiment 2. However, the results of our re-instantiation analyses (see below) imply that item-specific information may nonetheless be present in PPA during imagery.

### Evidence of perceptual pattern re-instantiation during imagery by ROI

**Figure 4:**
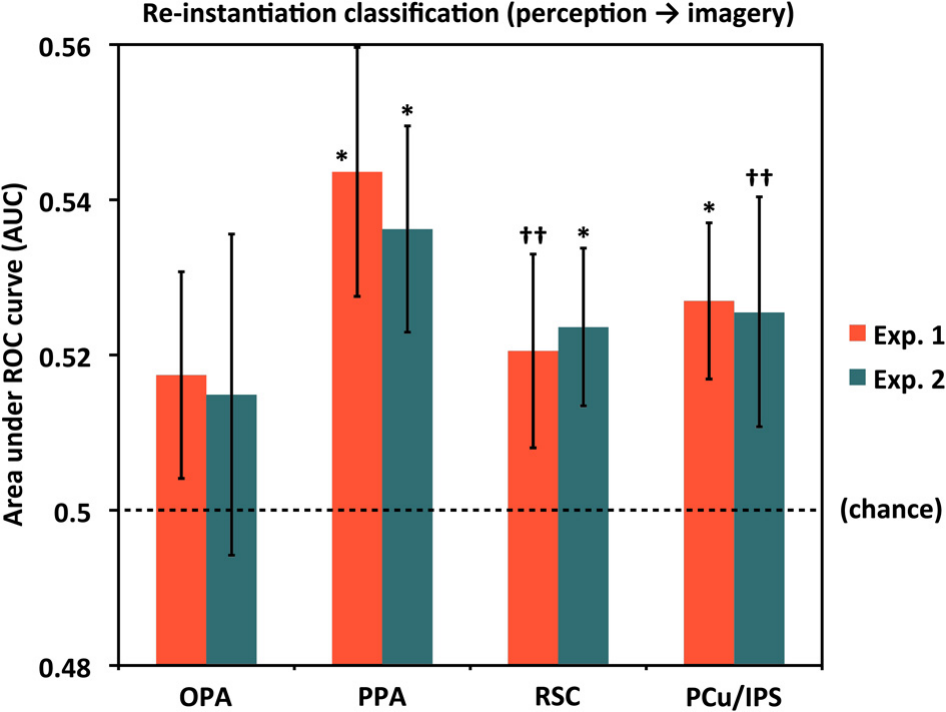
**Re-instantiation classification accuracy for distinguishing between individual scenes during mental imagery by ROI**. For these analyses, classifiers were trained with perception trials and tested on imagery trials, whereas the results shown in Figure 3B were both trained and tested with subsets of the imagery trials. PPA, RSC, and PCu/IPS all exhibited re-instantiation accuracies that were above chance (AUC = 0.5), either significantly or at a trend level, in one or both experiments. OPA re-instantiation accuracies were numerically but not significantly above chance in both experiments. Analyses used 80 voxels per hemisphere per region, for a total of 160 voxels per region. ^*^p < 0.05, ^††^*p* < 0.13. Error bars represent s.e.m. See text and Table 1 for full statistics.

We next tested for evidence of re-instantiation (similar item-specific neural activity during perception and imagery) in individual ROIs using a set of classifiers given the *Perceive* conditions as training data and the corresponding *Image* conditions as test data (see Materials and Methods). Results for these re-instantiation analyses in each ROI are shown in Figure 4 and Table 1C. Although classifier accuracies in these analyses for the OPA were numerically above chance, the difference was not significant in either Experiment 1 (AUC = 0.517) or Experiment 2 (AUC = 0.515). However, re-instantiation classification in the other ROIs exhibited significant performance above chance in either Experiment 1 (AUCs: PPA = 0.544, PCu/IPS = 0.527) or Experiment 2 (AUCs: PPA = 0.536, RSC = 0.524) or both, with weaker trends for RSC in Experiment 1 [AUC = 0.521 (*p* = 0.12)] and PCu/IPS in Experiment 2 [AUC = 0.525 (*p* = 0.11)].

Notably, in PPA the re-instantiation analyses were significantly better than chance in both experiments whereas cross-validation imagery classification was significant only at a trend level in Experiment 1, and not significantly different from chance in Experiment 2. This suggests that stimulus-specific information may indeed be present in PPA during mental imagery. One possibility for why item-specific information was not detected for imagery classification could be that item-specific information in PPA during imagery is more variable than in other areas (e.g., perhaps due to the particular features participants focus on for different imagery trials) but nonetheless consistently reflects some portion of activity patterns exhibited during perception, which are presumably more stable from trial to trial than imagery-related patterns. Such a situation would reduce cross-validation performance from imagery trials to imagery trials, while sparing performance on perception-to-imagery classification.

### Classifying perception vs. imagery

**Figure 5:**
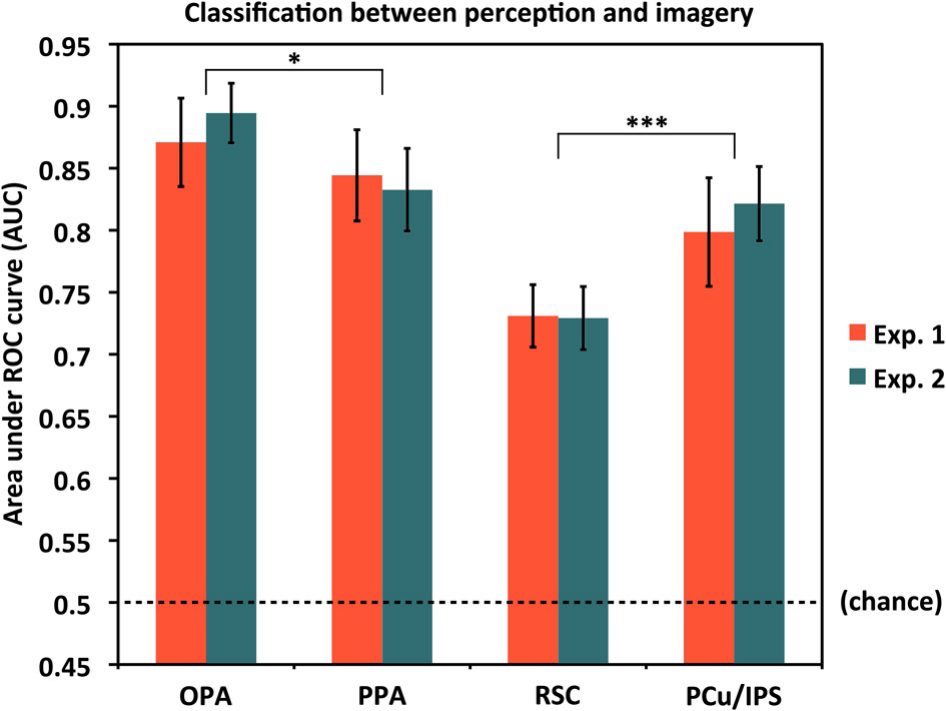
**Classification accuracy for distinguishing between the overall processes of perception and mental imagery by ROI**. In all cases, accuracies were significantly above chance (AUC = 0.5), but there were significant differences in accuracy by region. OPA differentiated between perception and imagery the best, followed by PPA, PCu/IPS, and RSC. Pairwise comparisons between OPA and PPA, and between PCu/IPS and RSC, were significant, though PPA and PCu/IPS did not significantly differ. Analyses used 80 voxels per hemisphere per region, for a total of 160 voxels per region. ^*^p < 0.05, ^***^p < 0.001. Error bars represent s.e.m. See text and Table 1 for full statistics.

We also asked to what extent the classifier was able to distinguish perception trials from imagery trials on the whole, regardless of the specific items being seen or visualized. As noted above, for this analysis, we coded each trial as either a *Perception* or *Imagery* trial and used a single cross-validation classifier. Results are shown in Figure 5. As expected, performance for classifying perception vs. imagery was high, and significantly above chance in all ROIs and both experiments (all AUC > 0.72, all *p* < 10^−5^). However, perception vs. imagery classification differed by area in both Experiment 1 [*F*_(3, 45)_ = 13.79, *p* = 1.64 × 10^−6^] and Experiment 2 [*F*_(3, 33)_ = 15.95, *p* = 1.40 × 10^−6^; both One-Way repeated-measures ANOVAs], supporting previous hypotheses that different areas along the visual processing pipeline for scenes may not all distinguish equally between perceptual and reflective processing (Johnson et al., 2007; Johnson and Johnson, 2009). OPA distinguished the most between perception and imagery, significantly more so than PPA [AUCs: 0.881 vs. 0.839, *t*_(27)_ = 2.77, *p* = 0.010]; PPA did not significantly differ from PCu/IPS [AUCs: 0.839 vs. 0.808, *t*_(27)_ = 1.55, *p* = 0.13]; but PCu/IPS distinguished between perception and imagery significantly more than RSC [AUCs: 0.808 vs. 0.730, *t*_(27)_ = 3.71, *p* = 0.00095; values were collapsed across experiment for these comparisons, as the label modality (visual or auditory) should not be expected to affect perception vs. imagery classification].

### Classifying scene identity information in face-selective cortex

**Figure 6:**
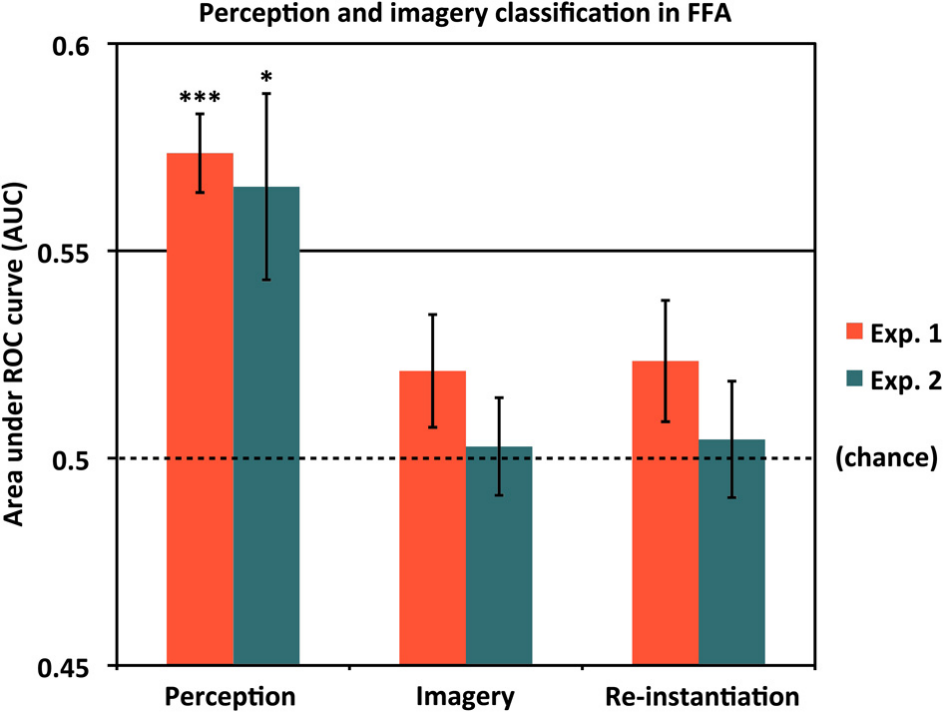
**Classifying scene identity information in face-selective cortex**. Classification accuracy for Experiments 1 and 2 using voxels from the fusiform face area (FFA). Results are shown for classifying between different scene items during perception (left bars), classifying between scene items during mental imagery (middle bars), and re-instantiation of perceptual information during mental imagery (right bars). Accuracies were significantly above chance (AUC = 0.5) during perception for both experiments, but did not differ from chance in either experiment during imagery or for re-instantiation. Analyses used 80 voxels from each of the left and right FFA, for a total of 160 voxels. ^*^p < 0.05, ^***^p < 0.001. Error bars represent s.e.m. See text and Table 1 for full statistics.

As our localizer data allowed us to isolate face-selective cortical areas in addition to scene-selective areas, we also addressed the question of whether voxels selective for non-scene categories nevertheless contained information about scene identity during perception and/or mental imagery. Results are shown in Figure 6 and Table 1. Notably, even after choosing the most face-selective voxels in the FFA, we still found significantly above-chance classification between scene items during perception in both Experiment 1 (AUC = 0.574) and Experiment 2 (AUC = 0.565). However, classification between scene items during imagery did not significantly differ from chance in either Experiment 1 [AUC = 0.521 (*p* = 0.14)] or Experiment 2 [AUC = 0.503 (n.s.)], nor did re-instantiation classification [Experiment 1: AUC = 0.523 (*p* = 0.13); Experiment 2: AUC = 0.505 (n.s.)]. In both experiments, classification between scene items was significantly better during perception than during imagery [Experiment 1: *t*_(15)_ = 4.41, *p* = 0.00050; Experiment 2: *t*_(11)_ = 2.55, *p* = 0.027]. Thus, even the most face-selective voxels in the FFA represent information distinguishing individual scenes during perception. We did not find strong evidence of FFA representing scene identity information during imagery (although there was a very weak trend in that direction in Experiment 1), but of course it is still possible that more sensitive experiments could uncover such information. However, even if scene identity information does exist in FFA during imagery, the current findings suggest that it is present to a smaller degree than in our scene-selective ROIs, or in the FFA itself during perception.

### Replication

In addition to summarizing AUCs, *t*-statistics, *p*-values, and effect sizes (Cohen's d) for the critical results presented above, Table 1 also presents *X*^2^ and *p*-values for the two experiments combined, using Fisher's method of combining *p*-values across replications (Fisher, 1925). Although Experiment 2 was initially conceived as a control experiment to confirm that the visual labels used in Experiment 1 did not drive successful classification during mental imagery, it is clear from the data that Experiment 2 replicated Experiment 1 very closely, and in many cases AUCs and effect sizes were greater for Experiment 2 than Experiment 1. Thus, given no evidence that visual vs. auditory labels made a difference in the results of the two experiments, we viewed it as appropriate to treat these experiments as a two-study meta-analysis and combine their *p*-values.

Considering these combined *p*-values also does not substantially alter the interpretation of any major results, but it does afford even greater confidence that the results obtained in each study individually were not due to random sampling fluctuations. Using the meta-analysis *p*-values, classification of item-specific information during perception was significantly above chance in all ROIs (including FFA); classification of item-specific information during imagery was significantly above chance in OPA, RSC, and PCu/IPS (but not PPA or FFA); and re-instantiation classification was significantly above chance in PPA, RSC, and PCu/IPS (but not OPA or FFA).

### Contributions of mean activation

In MVPA, it can be important to consider to what extent differences between conditions simply reflect difference in overall activation levels and not the “pattern” of activity in a region *per se* (e.g., Coutanche, 2013). To address this question, we performed three control analyses, each repeating the analysis above with a transformed version of the data. One such analysis considered the original data with the mean activation value (across voxels, within each trial) subtracted out (“mean-subtracted”); one considered *only* the mean activation value as the sole feature input into classification (“mean-only”); and one considered the original data after Z-scoring across voxels within each trial (“Z-scored”), which also has the effect of removing the mean activation value.

Full results from these control analyses are presented in Supplementary Table 1. Generally speaking, the pattern of results suggested that mean activation values were not a critical constituent of the successful classification performance in the analyses presented above. Although mean activation values were occasionally informative (i.e., performance of the mean-only classification was above chance), the mean-only classification was often at chance in cases where the original-data classification was successful, and even when the mean-only classification was above chance, its performance was almost always poorer than the original-data classification.

Furthermore, consideration of the mean-subtracted and Z-scored analyses showed that their performance was very similar to that of the original-data classification. In some instances, the mean-subtracted or Z-scored data produced slightly better performance than the original data and in other instances they were slightly worse, but overall, differences were essentially negligible. This demonstrates that even in cases where the mean activation value was informative, it did not generally convey a significant amount of unique information (i.e., information that was not also encoded in the activity patterns of the mean-subtracted or Z-scored data).

## Discussion

### Item-specific activity in scene-selective areas during perception and imagery

In this study, we found that item-specific scene information was present in multiple scene-selective cortical areas during both visual perception and visual mental imagery. This finding supports and extends previous work that has found sub-category-level information represented in various regions of scene-selective CSE cortex during perception (Kriegeskorte et al., 2007; Walther et al., 2009; Park et al., 2011; Bonnici et al., 2012; Epstein and Morgan, 2012), as well as work that has uncovered item-specific information in other areas during visual mental imagery (Thirion et al., 2006; Harrison and Tong, 2009; Serences et al., 2009; Stokes et al., 2009; Reddy et al., 2010; Lee et al., 2012; Xing et al., 2013). However, to our knowledge, this is the first study demonstrating that item-specific information about natural scenes is represented in multiple areas of scene-selective cortex during reflective processes engaged for mental imagery. This result, combined with the results from our perception-to-imagery re-instantiation analyses, provides additional evidence in favor of models that claim information relevant to the item held in mind is represented in CSE visual areas during reflective processing, and furthermore that this activity supports reflection by partially re-instantiating the same patterns of neural activity that were experienced when the item was initially perceived (Petrides, 1994; Kosslyn et al., 2001; Curtis and D'Esposito, 2003; Ruchkin et al., 2003; Pasternak and Greenlee, 2005; Ranganath and D'Esposito, 2005; Johnson et al., 2007).

When considering activity from all of our scene-selective ROIs combined (Figure 2), the evidence in favor of item-specific activity during both perception and imagery, and re-instantiation from perception to imagery, was clear; all analyses in the “Combined” region (Table 1) demonstrated large effect sizes with strong statistical significance. Classifier performance was less strong in the individual scene-selective ROIs than in the combined region, suggesting that individual ROIs each contributed non-redundant information to the unified cross-region representation. However, it is notable that we still found some evidence of item-specific scene information in all individual ROIs during both perception and imagery. Future studies will no doubt be helpful for replicating (and extending) some of the borderline findings reported here, but the present data demonstrate a promising start for the continued study of fine-grained information and how it is combined across regions in scene-selective cortex during both perception and imagery.

We also observed differences among regions that are consistent with previous observations and hypotheses, particularly with regard to how clearly different scene-selective areas distinguish between perception and imagery. It is, of course, reasonable to expect two areas to both represent information about visual scenes, but for the nature of that information to differ between the areas (e.g., Epstein, 2008; Park and Chun, 2009; Park et al., 2010). As expected, “higher” visual areas such as the RSC less reliably distinguished between perceiving and imagining scenes than the presumably “lower” level OPA area (with PPA and PCu/IPS falling in between), consistent with the hypothesis that areas later in the perceptual scene-processing pipeline may contain information at a higher level of abstraction that is more accessible and more readily re-instantiated during reflective processing, such as retrieving and/or reactivating information during mental imagery or refreshing active representations (Johnson et al., 2007; Johnson and Johnson, 2009). Future studies will be needed to determine if classification accuracy in different areas can be manipulated experimentally by varying the type and degree of low-level or high-level information differentiating scene exemplars.

As noted in the Introduction, several previous studies have used MVPA to examine the representation of visual information during perception in scene-selective cortex at the category, sub-category, and exemplar levels. Notably, Bonnici et al. (2012) demonstrated that it is possible to decode highly similar natural scenes at the exemplar level during perception. In this study, however, we opted to use scene exemplars that were drawn from different scene sub-categories, to maximize our chances of success for imagery-based decoding. This allowed us to conclude with confidence that scene identity information can be decoded from activity in scene-selective extrastriate cortex for exemplars with relatively large differences in low-level image features, but leaves open the question as to whether more fine-grained differences (e.g., between two highly similar beach exemplars) could also be decoded during mental imagery. Future studies could extend our design to include imagery of exemplars drawn from the same scene sub-categories to address this question.

It is also worth noting that although studies such as those by Walther et al. (2009) and Park et al. (2011) have demonstrated successful classification between scene sub-categories, it is still unknown whether semantically labeled sub-categories (e.g., “beaches” vs. “deserts”) truly enjoy a privileged categorical representation in visually responsive cortex. An alternative hypothesis is that scene sub-categories (beaches/deserts) and within-sub-category exemplars (beach 1/beach 2) are differentiated using the same set of low-level visual features, and that grouping scene stimuli by a semantic category label simply tends to produce collections of stimuli that are clustered closely enough on those feature dimensions (and far enough from the collections produced from other semantic labels) to aid classification. Thus, what distinguishes two scenes from different sub-categories, vs. what distinguishes two scenes within the same sub-category, may not itself be a categorical distinction, but instead only a difference of degrees of featural similarity. Again, future MVPA studies of both perception and imagery, using scene stimuli with greater similarity and/or more explicitly defined low-level feature characteristics, could help address this question.

### Scene information in FFA

In addition to scene-selective areas, the present study also found that FFA encodes information differentiating individual scenes from one another during perception, but did not find any reliable indication that FFA represents item-specific scene information during imagery. This supports the finding of Park et al. (2011), who also found above-chance classification performance for sub-category-level scene information in FFA during perception. However, Park and colleagues' “urban” scene stimuli contained some representations of human beings, which they noted could have driven their results in FFA. In contrast, our scene stimuli contained no representations of human or animal life, and thus our study resolves the ambiguity over whether scene information alone, devoid of faces or bodies, can drive above-chance classification in FFA during perception.

Although FFA has been repeatedly shown to activate more for faces than for other categories of visual stimuli, it does not activate *exclusively* for faces; other categories, including scenes, do activate the FFA above baseline, even if the magnitude of that activation is less than for faces (e.g., Kanwisher et al., 1997, 1999; McCarthy et al., 1997; Gauthier et al., 2000; Tong et al., 2000; Yovel and Kanwisher, 2004). Our results thus suggest that this activity evoked in FFA by non-face stimuli does carry information about those stimuli's identities; however, it remains to be shown whether this information is actually used by the brain in scene identification. At the same time, if the FFA is involved to some extent in natural scene processing during perception, these results could partially help explain the navigation deficits that can accompany both acquired and congenital prosopagnosia, although both forms of prosopagnosia are rather heterogeneous disorders that may implicate a variety of visual deficits and brain areas depending on the patient in question (Duchaine and Yovel, 2008).

It is also notable that although we observed scene-specific activity in FFA during perception, we found no such evidence during mental imagery. Although it is possible that FFA does contain relatively small amounts of item-specific information for scenes during imagery that were simply too weak to be detected, another possibility is that FFA processes certain features of all incoming perceptual stimuli in a way that can be read out by fMRI-based classification analyses, but that this information is not used or re-instantiated during mental imagery of scenes. PPA also showed relatively weak performance, compared to other scene-selective regions, in the classification of individual scene representations during imagery, but a key difference is that PPA showed substantially stronger performance in the re-instantiation analyses whereas FFA did not. Future studies employing more stimulus categories, more ROIs, and more trials will be needed to address the questions of whether other category-selective areas besides FFA represent information about the identities of stimuli outside their preferred category during perception (or even imagery), whether FFA contains identity information about non-face stimuli during imagery to a degree that was not detectable in the present investigation, and what factors may influence classification success for scene identity in PPA and other scene-selective regions during perception and/or imagery.

### Statistical and methodological considerations

Results in the analyses classifying over all scene areas were very robust for this area of research, with all AUCs > 0.55 and *p* < 0.01 in the imagery and re-instantiation analyses, and even stronger during perception. The classification AUC values for individual ROIs tended to be lower (e.g., many around 0.53–0.54, with chance = 0.50 and perfect classification = 1.0). However, it is important to consider several important factors when interpreting the magnitude of such findings. First, there are many different configurations of classification algorithms and parameters to choose from, which will tend to yield varying results. The different methods should agree in broad terms, but some might yield higher raw classification values on average, with the drawback of greater between-subject variability that would lead to decreased statistical significance overall. In this study, we opted to use a more conservative algorithm (SVM) and method of reporting its results (area under ROC curve) that in our previous tests had lower variance than other methods, even if the mean performance values were not the highest.

These values are also highly consistent with those reported by similar previous studies. For example, Eger et al. (2008) obtained only about 55% accuracy (chance = 50%) classifying exemplars of objects in the LOC during perception, and one might expect classification accuracy during imagery to be a bit lower than during perception (as we indeed found here). Comparable performance was found by Bonnici et al. (2012) for classifying between scene exemplars during perception based on activity in parahippocampal gyrus. Lee et al. (2012), whose experiment design is similar to the one reported here, also reported classification accuracy of just a few percentage points above chance for imagery of objects based on activity in object-selective cortex. Although it is difficult to make direct comparisons across studies given the heterogeneity of visual information studied, brain regions examined, analysis techniques used, output measures reported, fMRI parameters applied, statistical power obtained (numbers of participants and scan time per participant), and experimental designs used (e.g., block vs. event-related designs), it is clear that low classification accuracies are common for research of this sort, but nonetheless consistent enough to yield statistically significant results with typical participant sample sizes.

Because classifier performance values vary between algorithms and studies, it may be useful to consider the values of standard effect-size measures such as Cohen's d (see Table 1). For example, for classification of item-level information during mental imagery in individual scene-selective regions, all the results we reported as significant (*p* < 0.05) had effect sizes between 0.566 and 0.927. These would generally be considered medium- to large-sized effects (Cohen, 1988), even though the corresponding AUC values for those effects were only 0.536 and 0.554, respectively.

We also note that all of the *p*-values reported here are two-tailed, to err on the side of being conservative, although the use of one-tailed values could be justified. Researchers continue to debate over when and whether one-tailed tests should be used; but when this issue was heavily discussed in the 1950s, Kimmel (1957) stated three criteria for appropriate use of one-tailed tests: (1) “… when a difference in the unpredicted direction, while possible, would be psychologically meaningless.” (2) “… when results in the unpredicted direction will, under no conditions, be used to determine a course of behavior different in any way from that determined by no difference at all.” (3) “… when a directional hypothesis is deducible from psychological theory but results in the opposite direction are not deducible from coexisting psychological theory.” These conditions would seem to be satisfied in the case of an algorithm that either performs better than chance when given meaningful input or exactly at chance (on average) when given random input. Any accuracies/AUCs dipping below the 0.5 chance threshold can only denote performance which is at chance, but which has a value less than 0.5 simply due to random sampling fluctuations. As the only neurally/psychologically viable interpretations are of performance above chance or a null result, a one-tailed test would be appropriate by Kimmel's criteria. Thus, all the *p*-values reported here could potentially be cut in half; although this would not substantially change any major results, it would bring several individual analyses currently labeled “trends” within the conventional 0.05 significance threshold.

Another methodological issue worthy of consideration is the possible contribution of eye movements to our results. In the present study, we did not monitor eye movements in the scanner or instruct participants to maintain fixation on a single point during imagery or perception, which invites the question as to how classification performance might be affected by requiring participants to maintain fixation. One possibility is that requiring fixation could reduce trial-to-trial variability and thus improve classifier performance, either from lesser variability in bottom-up visual input or in the cognitive strategies employed by participants to perform mental imagery, or both. On the other hand, maintaining fixation is generally more effortful and less natural than free-viewing. Therefore, it is also possible that requiring fixation may split participants' attention between performing the actual task and their efforts to maintain a steady eye position, and as a result actually reduce the quality of perceptual and imagined representations and thus reduce classification performance.

Previous investigations of receptive-field sizes in the areas we examined suggest that they are typically large and thus fairly robust to changes in eye position. Specifically, Oliva and Torralba (2006) noted that “Receptive fields in the inferior temporal cortex and parahippocampal region cover most of the useful visual field (20–40°)” (p. 34). Similarly, MacEvoy and Epstein (2007) found that receptive fields in the PPA, RSC, and OPA even spanned across visual hemifields and concluded that these areas “may support scene perception and navigation by maintaining stable representations of large-scale features of the visual environment that are insensitive to the shifts in retinal stimulation that occur frequently during natural vision” (p. 2089). Such receptive fields would typically cover the entirety of the stimuli we presented (around 20° of visual angle), and thus making saccades within the bounds of those stimuli should, in theory, have little effect on activity patterns in those regions. A follow-up study specifically examining the consequences of manipulating fixation requirements would be necessary to resolve these questions conclusively, but based on the studies of receptive field sizes cited above, we would predict the effect of fixation vs. free-viewing on classification performance, if any, to be relatively modest.

## Summary

Overall, the present study presents strong evidence that several scene-selective extrastriate areas represent individuating information about complex natural scenes during both perception and the reflective processes involved in mental imagery, and furthermore that neural activity produced during scene perception is re-instantiated in scene-selective cortical areas in the service of reflective thought. Furthermore, we again find that certain scene-selective regions differentiate more than others between the overall processes of perception and reflection. We also found that item-specific scene information is present in the face-selective FFA during perception, but found no evidence that FFA represents scene identity information during top-down reflective processing such as mental imagery. Future work will be needed to more precisely establish the nature of the information represented in each cortical area during perception and/or imagery, how that information differs between areas, whether more fine-grained information identifying exemplars within scene sub-categories may also be successfully decoded during mental imagery, what factors may contribute to which and how much perceptual information is successfully re-instantiated during reflective thought, how specificity of perceptual and reflective representations may vary in different subject populations, and how information in various regions contributes to distinguishing between perception and reflection.

## Author contributions

Matthew R. Johnson co-designed the experiments; collected the data; performed primary data analyses; created the figures; and co-wrote the text. Marcia K. Johnson co-designed the experiments; co-wrote the text; and advised on all aspects of the research.

### Conflict of interest statement

The authors declare that the research was conducted in the absence of any commercial or financial relationships that could be construed as a potential conflict of interest.

## Supplementary material

Supplementary Figure 1 **Comparison of classification using different numbers of voxels per region of interest**. Classification analyses for individual scene-selective ROIs in the main text (Figure 3) used 80 voxels per ROI per hemisphere, for a total of 160 voxels per ROI. Here, those analyses are repeated using 10, 20, 40, 80, 160, or 320 voxels per ROI per hemisphere. If a participant did not have enough in-brain voxels in a given ROI, all of their in-brain voxels in a 10 mm radius were used, so some analyses contain fewer voxels than the stated number for some participants. Classification performance varied with region, condition, and experiment, but in most cases performance reached a plateau by 80 voxels per ROI per hemisphere, and in some cases performance worsened at higher voxel counts (e.g., in OPA for imagery classification), likely due to the inclusion of white matter or other noise voxels. *P*-values represent uncorrected two-tailed *t*-tests against chance (0.5) at each point, color-coded according to experiment. Error bars represent s.e.m.

Supplementary Table 1 **Contributions of mean activation levels to classifier performance**. All *p*-values represent two-tailed *t*-tests against a chance AUC value of 0.5. For each region, experiment, and type of analysis, classifier performance is reported for the original data (as reported in the main manuscript and Table 1), the data with the mean activation value (across voxels, within each trial) subtracted out, a classifier based *only* on mean activity levels, and the data after Z-scoring across features (within each trial). Experiment 1: all degrees of freedom (*df*) = 15. Experiment 2: all *df* = 11. AUC = area under ROC curve.
