# Supplementary figures and images for: Decoding individual natural scene representations during perception and imagery
Source: Front Hum Neurosci. 2014 Feb 12;8:59. doi: 10.3389/fnhum.2014.00059 (PMC3921604; doi:10.3389/fnhum.2014.00059)

# Perceive

# Image

OPA

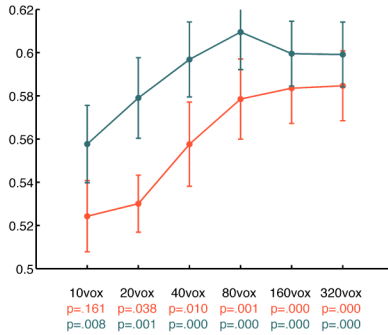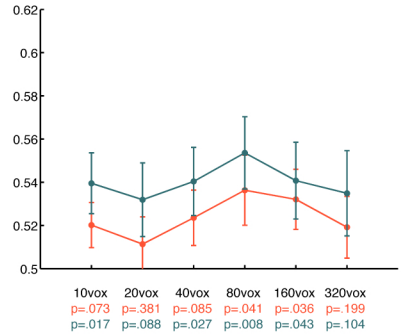

PPA

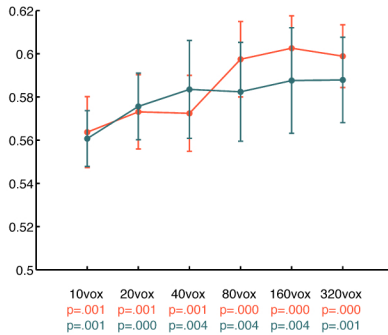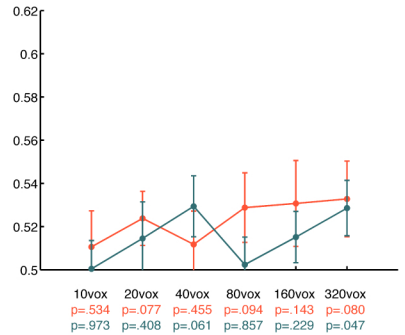

RSC

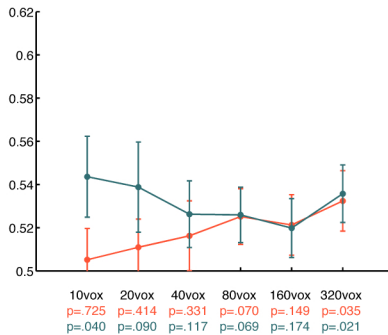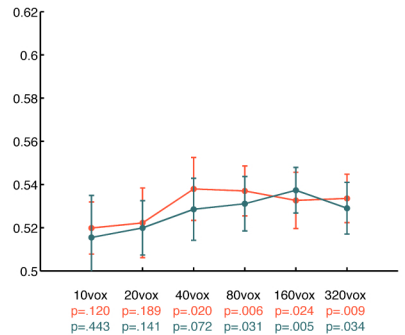

PCu/IPS

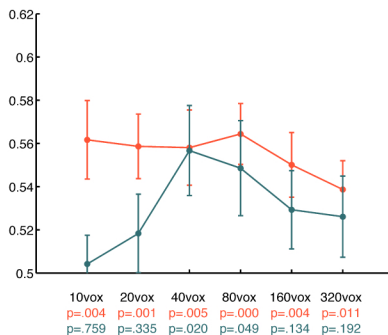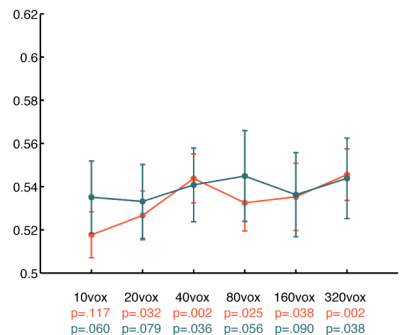

Experiment 1

Experiment 2

Supplement: Supplementary Figure 1 — Comparison of classification using different numbers of voxels per region of interest. Classification analyses for individual scene-selective ROIs in the main text (Figure 3) used 80 voxels per ROI per hemisphere, for a total of 160 voxels per ROI. Here, those analyses are repeated using 10, 20, 40, 80, 160, or 320 voxels per ROI per hemisphere. If a participant did not have enough in-brain voxels in a given ROI, all of their in-brain voxels in a 10 mm radius were used, so some analyses contain fewer voxels than the stated number for some participants. Classification performance varied with region, condition, and experiment, but in most cases performance reached a plateau by 80 voxels per ROI per hemisphere, and in some cases performance worsened at higher voxel counts (e.g., in OPA for imagery classification), likely due to the inclusion of white matter or other noise voxels. P-values represent uncorrected two-tailed t-tests against chance (0.5) at each point, color-coded according to experiment. Error bars represent s.e.m. [file Presentation1.PDF]
