# Supplementary material for: Decoding individual natural scene representations during perception and imagery
Source: Front Hum Neurosci. 2014 Feb 12;8:59. doi: 10.3389/fnhum.2014.00059 (PMC3921604; doi:10.3389/fnhum.2014.00059)
Supplement: Supplementary Table 1 — Contributions of mean activation levels to classifier performance. All p-values represent two-tailed t-tests against a chance AUC value of 0.5. For each region, experiment, and type of analysis, classifier performance is reported for the original data (as reported in the main manuscript and Table 1), the data with the mean activation value (across voxels, within each trial) subtracted out, a classifier based only on mean activity levels, and the data after Z-scoring across features (within each trial). Experiment 1: all degrees of freedom (df) = 15. Experiment 2: all df = 11. AUC = area under ROC curve. [file DataSheet1.PDF]

Supplementary Table 1. Contributions of mean activation levels to classifier performance. All p-values represent two-tailed t-tests against a chance AUC value of 0.5. For each region, experiment, and type of analysis, classifier performance is reported for the original data (as reported in the main manuscript and Table 1), the data with the mean activation value (across voxels, within each trial) subtracted out, a classifier based *only* on mean activity levels, and the data after Z-scoring across features (within each trial). Experiment 1: all degrees of freedom (df) = 15. Experiment 2: all df = 11. AUC = area under ROC curve.

A. Classification of item-specific scene information during perception

	**Experiment 1**		**Experiment 2**	
**ROI**	**AUC**	**p**	**AUC**	**p**
**OPA** (original data):	.579	.00071	.610	$5.6x10^{-5}$
- mean subtracted out:	.583	.00091	.623	$5.0x10^{-5}$
- mean-only classifier:	.489	.43	.514	.18
- Z-scored across features:	.580	.0015	.618	$4.0x10^{-5}$
**PPA** (original data):	.598	$4.9x10^{-5}$	.583	.0041
- mean subtracted out:	.595	.00011	.580	.0047
- mean-only classifier:	.534	.022	.557	.0016
- Z-scored across features:	.596	.00011	.579	.0039
**RSC** (original data):	.525	.069	.526	.067
- mean subtracted out:	.520	.12	.528	.050
- mean-only classifier:	.524	.024	.495	.72
- Z-scored across features:	.521	.10	.530	.045
**PCu/IPS** (original data):	.564	.00038	.548	.051
- mean subtracted out:	.568	.00058	.557	.020
- mean-only classifier:	.494	.58	.512	.30
- Z-scored across features:	.563	.00040	.559	.017
**Combined** (original data):	.627	$9.1x10^{-6}$	.634	$8.3x10^{-5}$
- mean subtracted out:	.633	$1.1x10^{-5}$	.647	$7.1x10^{-5}$
- mean-only classifier:	.500	.97	.513	.37
- Z-scored across features:	.631	$1.9x10^{-5}$	.651	$3.9x10^{-5}$
**FFA** (original data):	.574	$1.3x10^{-6}$	.565	.014
- mean subtracted out:	.573	$6.6x10^{-6}$	.567	.014
- mean-only classifier:	.521	.13	.507	.72
- Z-scored across features:	.572	$4.4x10^{-6}$	.568	.012

B. Classification of item-specific scene information during imagery

	Experiment 1		Experiment 2	
**ROI**	**AUC**	**p**	**AUC**	**p**
**OPA** (original data):	.536	.042	.554	.0083
- mean subtracted out:	.538	.022	.555	.0087
- mean-only classifier:	.495	.639	.519	.12
- Z-scored across features:	.537	.034	.559	.0039
**PPA** (original data):	.529	.094	.503	.85
- mean subtracted out:	.525	.15	.501	.96
- mean-only classifier:	.500	.98	.517	.21
- Z-scored across features:	.529	.11	.500	.98
**RSC** (original data):	.537	.0057	.531	.031
- mean subtracted out:	.535	.0093	.522	.15
- mean-only classifier:	.479	.077	.499	.97
- Z-scored across features:	.537	.0048	.524	.11
**PCu/IPS** (original data):	.533	.025	.545	.055
- mean subtracted out:	.535	.016	.550	.041
- mean-only classifier:	.499	.91	.519	.084
- Z-scored across features:	.534	.011	.550	.038
**Combined** (original data):	.560	.0023	.558	.0064
- mean subtracted out:	.558	.0058	.561	.0063
- mean-only classifier:	.493	.44	.530	.015
- Z-scored across features:	.557	.0053	.567	.0029
**FFA** (original data):	.521	.14	.503	.82
- mean subtracted out:	.524	.069	.508	.50
- mean-only classifier:	.485	.27	.484	.40
- Z-scored across features:	.523	.071	.511	.37

C. Re-instantiation of item-specific information from perception to imagery

	Experiment 1		Experiment 2	
**ROI**	**AUC**	**p**	**AUC**	**p**
**OPA** (original data):	.517	.21	.515	.49
- mean subtracted out:	.520	.13	.512	.57
- mean-only classifier:	.501	.88	.510	.62
- Z-scored across features:	.521	.11	.514	.54
**PPA** (original data):	.544	.016	.536	.020
- mean subtracted out:	.542	.021	.531	.035
- mean-only classifier:	.543	.0037	.528	.023
- Z-scored across features:	.539	.022	.533	.039
**RSC** (original data):	.521	.12	.524	.040
- mean subtracted out:	.518	.17	.525	.036
- mean-only classifier:	.522	.017	.517	.31
- Z-scored across features:	.518	.20	.524	.036
**PCu/IPS** (original data):	.527	.017	.525	.11
- mean subtracted out:	.524	.043	.532	.034
- mean-only classifier:	.523	.032	.515	.21
- Z-scored across features:	.526	.051	.529	.059
**Combined** (original data):	.553	.0083	.561	.0077
- mean subtracted out:	.552	.011	.560	.0096
- mean-only classifier:	.522	.063	.513	.34
- Z-scored across features:	.551	.0098	.558	.014
**FFA** (original data):	.523	.13	.505	.75
- mean subtracted out:	.523	.13	.509	.54
- mean-only classifier:	.521	.052	.504	.73
- Z-scored across features:	.523	.14	.507	.61

D. Classification between the overall processes of perception and mental imagery

	Experiment 1		Experiment 2	
**ROI**	**AUC**	**p**	**AUC**	**p**
**OPA** (original data):	.870	$3.2x10^{-8}$	.893	$5.0x10^{-9}$
- mean subtracted out:	.870	$2.8x10^{-8}$	.893	$4.9x10^{-9}$
- mean-only classifier:	.779	$4.7x10^{-8}$	.796	$9.3x10^{-8}$
- Z-scored across features:	.867	$3.5x10^{-8}$	.889	$4.4x10^{-9}$
**PPA** (original data):	.844	$1.2x10^{-7}$	.831	$8.6x10^{-7}$
- mean subtracted out:	.833	$2.6x10^{-7}$	.822	$8.9x10^{-7}$
- mean-only classifier:	.812	$5.8x10^{-9}$	.803	$1.7x10^{-8}$
- Z-scored across features:	.835	$2.4x10^{-7}$	.820	$6.8x10^{-7}$
**RSC** (original data):	.729	$1.7x10^{-7}$	.727	$2.4x10^{-6}$
- mean subtracted out:	.722	$2.9x10^{-7}$	.726	$1.4x10^{-6}$
- mean-only classifier:	.568	.019	.599	.00012
- Z-scored across features:	.724	$4.4x10^{-7}$	.731	$1.3x10^{-6}$
**PCu/IPS** (original data):	.798	$5.5x10^{-6}$	.820	$4.0x10^{-7}$
- mean subtracted out:	.801	$4.8x10^{-6}$	.826	$1.9x10^{-7}$
- mean-only classifier:	.597	.00086	.616	.0033
- Z-scored across features:	.798	$4.7x10^{-6}$	.814	$3.1x10^{-7}$
**Combined** (original data):	.914	$2.9x10^{-10}$	.940	$2.9x10^{-11}$
- mean subtracted out:	.914	$2.5x10^{-10}$	.943	$1.0x10^{-11}$
- mean-only classifier:	.726	$3.2x10^{-7}$	.747	$3.9x10^{-8}$
- Z-scored across features:	.912	$2.3x10^{-10}$	.941	$1.6x10^{-11}$
**FFA** (original data):	.771	$3.3x10^{-7}$	.762	$2.0x10^{-5}$
- mean subtracted out:	.771	$2.7x10^{-7}$	.762	$1.8x10^{-5}$
- mean-only classifier:	.623	.0024	.569	.023
- Z-scored across features:	.764	$3.5x10^{-7}$	.764	$1.2x10^{-5}$
